# Effects of Palm Stearin versus Butter in the Context of Low-Carbohydrate/High-Fat and High-Carbohydrate/Low-Fat Diets on Circulating Lipids in a Controlled Feeding Study in Healthy Humans

**DOI:** 10.3390/nu13061944

**Published:** 2021-06-05

**Authors:** Parker N. Hyde, Teryn N. Sapper, Richard A. LaFountain, Madison L. Kackley, Alex Buga, Brandon Fell, Christopher D. Crabtree, Stephen D. Phinney, Vincent J. Miller, Sarah M. King, Ronald M. Krauss, William J. Kraemer, Jeff S. Volek

**Affiliations:** 1 Department of Human Sciences, The Ohio State University, 305 Annie & John Glenn Ave, Columbus, OH 43210, USA; parker.hyde@ung.edu (P.N.H.); bedell.387@osu.edu (T.N.S.); lafountainrich@gmail.com (R.A.L.); kackley.19@osu.edu (M.L.K.); buga.1@osu.edu (A.B.); brandon.fell@virtahealth.com (B.F.); crabtree.223@osu.edu (C.D.C.); vin.miller@gmail.com (V.J.M.); kraemer.44@osu.edu (W.J.K.); 2 Department of Kinesiology, University of North Georgia, Dahlonega, GA 30597, USA; 3 Virta Health, San Francisco, CA 94105, USA; steve@virtahealth.com; 4 Departments of Pediatrics and Medicine, University of California, San Francisco, CA 94609, USA; sarah.king@ucsf.edu (S.M.K.); ronald.krauss@ucsf.edu (R.M.K.)

**Keywords:** cholesterol, butter, palm oil, low-carbohydrate diet, saturated fat

## Abstract

Background. Foods rich in saturated fatty acids (SFAs) have been discouraged by virtue of their cholesterol-raising potential, but this effect is modulated by the food source and background level of carbohydrate. Objective. We aimed to compare the consumption of palm stearin (PS) versus butter on circulating cholesterol responses in the setting of both a low-carbohydrate/high-fat (LC/HF) and high-carbohydrate/low-fat (HC/LF) diet in healthy subjects. We also explored effects on plasma lipoprotein particle distribution and fatty acid composition. Methods. We performed a randomized, controlled-feeding, cross-over study that compared a PS- versus a Butter-based diet in a group of normocholesterolemic, non-obese adults. A controlled canola oil-based ‘Run-In’ diet preceded the experimental PS and Butter diets. All diets were eucaloric, provided for 3-weeks, and had the same macronutrient distribution but varied in primary fat source (40% of the total fat). The same Run-In and cross-over experiments were done in two separate groups who self-selected to either a LC/HF (*n* = 12) or a HC/LF (*n* = 12) diet track. The primary outcomes were low-density lipoprotein-cholesterol (LDL-C), high-density lipoprotein (HDL)-C, triglycerides, and LDL particle distribution. Results. Compared to PS, Butter resulted in higher LDL-C in both the LC/HF (13.4%, *p* = 0.003) and HC/LF (10.8%, *p* = 0.002) groups, which was primarily attributed to large LDL I and LDL IIa particles. There were no differences between PS and Butter in HDL-C, triglycerides, or small LDL particles. Oxidized LDL was lower after PS than Butter in LC/HF (*p* = 0.011), but not the HC/LF group. Conclusions. These results demonstrate that Butter raises LDL-C relative to PS in healthy normocholesterolemic adults regardless of background variations in carbohydrate and fat, an effect primarily attributed to larger cholesterol-rich LDL particles.

## 1. Introduction

The impact of saturated fatty acid (SFA) consumption on health is one of the more contentious areas in nutrition with arguments both for [1,2] and against [3,4] limiting intake. An expert panel recently concluded that the evidence to limit saturated fat is weak and that higher intake of some SFA-rich foods (e.g., high-fat diary) are not associated with adverse cardiovascular outcomes [5]. The primary rationale for limiting SFA consumption is based on its cholesterol-raising potential, and the causative nature of LDL-C in cardiovascular disease (CVD) [6,7]. Although consuming SFA in place of other macronutrients increases LDL-C, the effects are variable [8], due in part to attributes that make up the food matrix. For example, palm oil which is relatively high in palmitic acid (C16:0), raises LDL-C and HDL-C when it is substituted for unsaturated fat sources [9,10] or stearic acid (C18:0), but decreases LDL-C compared with diets rich in myristic acid (C14:0)/lauric acid (C12:0) [11]. Since foods like butter and palm oil have a mix of SFAs varying in carbon length, as well as different positional distributions on the glycerol backbone that may affect cholesterol responses [12], predicting the net impact on LDL-C and other circulating lipids is not straightforward.

Another consideration is the lack of association between diet-induced changes in LDL-C and CVD hard outcome measures [13,14], implying there may be value in considering how SFA-containing foods impact other lipid markers. It is now recognized that high triglycerides and low HDL-C associate with a predominance of small, dense LDL particles that have greater atherogenic properties than larger LDL particles, including greater susceptibility to oxidation [15,16]. Moreover, higher abundance of even-chain SFAs (particularly palmitate) in plasma predicts increased risk of metabolic syndrome [17], type-2 diabetes [18,19], heart failure [20], and mortality [21]. How diets varying in different SFA-containing foods impact LDL particle distribution and accumulation of circulating SFAs has received little attention. 

Finally, the effects of consuming SFA-containing foods on circulating lipid responses are impacted by the carbohydrate to fat ratio of the diet [22,23,24,25]. Despite consuming 2–3 times more saturated fat, low-carbohydrate/high-fat (LC/HF) diets that promote enhanced fatty acid oxidation and decreased de novo lipogenesis (DNL) decrease circulating small LDL particles and fatty acid products of DNL [22,23,24], an effect that is independent of weight loss and LDL-C response [25]. 

The primary purpose of this study was to compare cholesterol responses to consumption of two SFA-based food sources (palm oil and butter) in healthy, normocholesterolemic individuals during weight-stable conditions. Although palm oil is more widely consumed and studied, we used palm stearin (PS), the solid fraction after additional fractionation of palm oil, because it more closely matches the total saturated fat content of butter. We used a randomized, cross-over, controlled-feeding study design that incorporated a controlled canola oil-based diet Run-In period. We replicated this experimental approach in the context of both LC/HF and high-carbohydrate/low-fat (HC/LF) diets. To provide in-depth assessment of the dietary effects beyond standard lipids, we assessed plasma lipoprotein particle subclasses, oxidized LDL, and fatty acid composition. 

## 2. Methods

### 2.1. Experimental Approach

**Figure 1:**
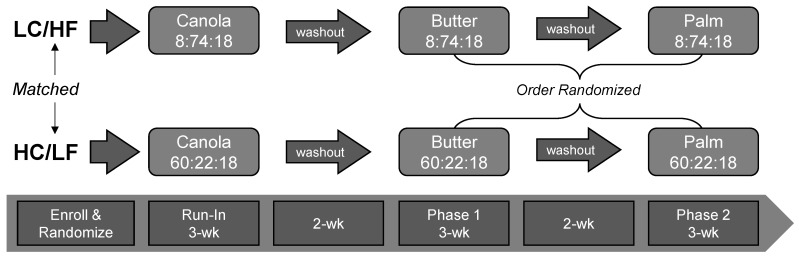
Experimental Approach. Subjects self-selected into the low-carbohydrate/high-fat (LC/HF) or high-carbohydrate/low-fat (HC/LF) diet group. Both groups were fed a 3-wk run-in diet high in canola oil. Baseline testing was performed before and following the canola oil diet phase. This was followed by two randomized and balanced experimental feeding phases that emphasized either butter or palm stearin, separated by a 2-week washout period. Numbers in the boxes with dietary fat source represent the percent energy from carbohydrate, fat and protein, respectively.

In order to compare the circulating cholesterol and fatty acid responses to PS and Butter in the context of both LC/HF and HC/LF diets, we enrolled healthy men and women who were habitually consuming a typical mixed moderate- to high-carbohydrate diet (verified by 3-day diet records). In order to reflect a ‘real-world application’ and minimize the number of cross-over periods, participants self-selected into either a LC/HF (*n* = 12) or HC/LF (*n* = 12) diet group. In order to stabilize subjects in both groups prior to being provided the experimental PS and Butter diets, we provided them with either a eucaloric LC/HF or HC/LF Run-In diet rich in canola oil. This Run-In period acted as a low saturated fat control that lasted three weeks and allowed subjects to become adapted to the background level of carbohydrate (low versus high). After the Run-In period, subjects within each group were provided PS- or Butter-based diets that each lasted 3-weeks separated by a 2-week washout period. The 2-week washout period allowed participants a break from the controlled feeding during which they were allowed to return to their habitual diet. The order of the PS and Butter diets was randomized via online randomization tools (www.randomizer.org) and balanced for each group (i.e., half started on PS and half started on the Butter diet) to control for possible order or carry-over effects. Testing occurred at baseline and after each of the three feeding phases (Figure 1).

### 2.2. Study Participants

Participants were recruited through posted flyers, word of mouth, and ResearchMatch through the Ohio State University (OSU) Center for Clinical and Translational Science. We recruited simultaneously for the LC/HF and HC/LF groups. Initially, a phone screening was performed to overview the study details and assess inclusion/exclusion criteria. “All subjects signed an informed consent document approved by the Institutional Review Board at the Ohio State University (#2015H0435, approved initially 6 July 2016)” Participants for both diet groups were between the ages of 21–65 years and non-obese (BMI 20–29 kg/m^2^). During screening, subjects completed medical, diet, and exercise history questionnaires. A fasting blood sample was obtained during screening and sent to Quest Diagnostics (Columbus, OH, USA) for a standard metabolic and lipid panels. Participants were required to be within normal ranges for standard metabolic panels and have total cholesterol < 250 mg/dL, LDL-C < 160 mg/dL, and triglycerides < 150 mg/dL. Individuals were excluded from participation if they had consumed a low-carbohydrate diet (<130 g/day) in the past 6-months or had a history of high blood pressure, diabetes, metabolic or endocrine dysfunction, gastrointestinal dysfunction, medications affecting lipids, food allergies/intolerances, and smoking, or were pregnant or lactating. A CONSORT diagram showing participant entry and passage through the study is provided in Supplementary Materials Figure S1.

### 2.3. Dietary Interventions

A 7-day rotational menu was developed for both the LC/HF and HC/LF diets using nutrient analysis software (Nutritionist Pro, Axxya Systems, Redmond, WA, USA). The menus were created using a base calorie level of 2500 kcal/day, which was adjusted for body size and estimated metabolic rate and scaled proportionately according to the participants’ calculated energy requirements. Caloric needs for each participant were determined using the Harris-Benedict Equation [26]. The menus for each participant were designed to be eucaloric and isonitrogenous throughout each feeding phase to maintain weight stability throughout the experimental period. The only ingredient that varied between each feeding phase was the single fat source of interest (canola oil, butter, and PS).

All food was weighed to the nearest 0.1 g to ensure accuracy of target nutrient goals for each diet. Daily protein intake (18%en) was constant across all feeding interventions with the only macronutrient difference being the ratio of carbohydrate to fat between the two groups. Within the LC/HF and HC/LF groups, the absolute amount of protein, carbohydrate and fat were the same during each diet phase with the main difference being the primary fat source (i.e., canola oil, butter, PS), which made up 40% of total fat calories. The primary fats were incorporated into meals in various ways including as a base of salad dressings, mixed in with dips, topping on steamed/roasted vegetables, and preparation of eggs. Because of the requirement to incorporate high amounts of the primary fat into meals, we emphasized leaner protein sources including egg whites. Mean nutrient intakes for each diet track (Table 1) and the fatty acid composition of the primary fat sources analyzed by gas chromatography (Covance, Princeton, NJ, USA) (Supplementary Materials Table S1) are reported. The Run-In diet was lower in SFA and higher in MUFA, especially oleic acid. Compared to the Butter diet, the PS diet was slightly lower in total SFA but had higher palmitic acid. An example meal plan for all controlled feeding periods is provided in Supplementary Materials Table S2.

Participants were provided with 100% of their caloric needs during each study intervention phase. All of the food was prepared in our metabolic kitchen using typical foods such as lean beef, chicken, salmon, egg whites, leafy greens, various starchy and non-starchy vegetables, fruits, and grain products. The use of certain foods varied by diet track to meet specific carbohydrate requirements. The meals were packaged with labels and picked up 3x/week to ensure food quality and safety. Participants were advised to avoid all other foods and beverages outside of what the study provided them except for a small list of very low/non-caloric products (i.e., coffee, tea, diet soda, pepper). In order to provide a break from the controlled-feeding, during the washout periods participants returned to their habitual diets. Subjects were repeatedly reminded during food pick-ups to maintain their normal activity level throughout all phases of the study, which was verbally verified.

### 2.4. Body Composition 

Body composition and bone mineral density analysis was completed using dual-energy x-ray absorptiometry (DXA) (iDXA, GE, Chicago, IL, USA). All measurements were conducted by a certified radiologist who specialized in this technology. Bone mineral density, total lean mass, and fat mass percentage were calculated using the iDXA. Height and weight were measured using a stadiometer and scale (SECA Model 703, Hamburg, Germany). Waist circumference measurements were taken by a trained researcher as defined by the World Health Organization (Gulick II, Fitness Mart, Gays Mills, WI, USA). Blood pressure was measured (Welch Allyn, Skaneateles Falls, NY, USA) by a trained researcher after participants had been seated quietly with feet on the floor for five minutes. After the first measurement the participant remained seated for an additional 2–3 min before a second measurement was taken. 

### 2.5. Fasting Blood Measures

All blood measures were obtained at screening, baseline, and after each diet phase. Fasting blood samples were obtained following an 8–12 h overnight fast via venipuncture in the antecubital fossa by a trained phlebotomist. Blood was collected into serum, serum separator, ethylenediaminetetraacetic acid (EDTA) and sodium heparin vacutainers. Plasma tubes were placed on ice while serum and serum separator tubes were allowed to sit at room temperature for 30 min prior to centrifugation to allow for clotting to occur. Whole blood was then centrifuged at 1500× *g* for 10 min at 4 °C. Fresh serum aliquots were sent to Quest Diagnostics (Columbus, OH, USA) for metabolic and lipid panel analysis. All remaining aliquots were snap-frozen in liquid nitrogen and stored at −80 °C until assay. Frozen samples for fatty acid composition, lipoproteins and oxidized LDL were kept frozen and only thawed once before analysis. Oxidized LDL and serum insulin were measured in duplicate via ELISA (Mercodia, Uppsala, Sweden and R&D Quantikine, Minneapolis, MN, USA, respectively). Inter- and intra-assay coefficients of variation were <10%.

### 2.6. Fatty Acid Composition

Phospholipids (PL) and triglycerides (TG) were extracted from serum samples with methanol, chloroform and water as our group has previously described [22,27]. In brief, the lipid extracts were separated on commercial silica gel G plates (AnalTech, Newark, DE, USA) and resulting fatty acid methyl esters were analyzed by gas chromatography (Shimadzu Corporation, Columbia, MD, USA). Authentic standards were used to identify fatty acids which were then quantitated with peak area and internal standards and reported as percent abundance. 

### 2.7. Plasma Lipoprotein Particle Analysis

As previously described [25] particle concentrations of very low-density lipoprotein (VLDL), intermediate-density lipoprotein (IDL), LDL, and HDL subfractions were analyzed in specific particle-size intervals using ion mobility, which uniquely allows for direct particle quantification as a function of particle diameter [28] following a procedure to remove other plasma proteins [29]. The ion mobility instrument utilizes an electrospray to create an aerosol of particles, which then pass through a differential mobility analyzer coupled to a particle counter. Particle concentrations (nmol/L) are determined for subfractions defined by the following size intervals (nm): VLDL: large (42.40–54.70), medium (33.50–42.39), small (29.60–33.49); IDL: large (25.00–29.59), small (23.33–24.99); LDL: large (22.0–23.32), medium (21.41–21.99), small (20.82–21.40), very small (18.0–20.81); HDL: large (10.50–14.50) and small (7.65–10.49). Inter-assay variation was reduced by inclusion of two in-house controls in each preparatory process and triplicate analysis. Coefficients of variation <15% for each subfraction measurement were maintained throughout. 

### 2.8. Statistical Analysis

The focus was on the comparison of PS to Butter with primary outcomes LDL-C, HDL-C, triglycerides, and LDL particles. We hypothesized higher LDL-C in Butter than PS. We estimated that the magnitude of difference (mean ± SD) would be approximately 10 ± 12 mg/dL, primarily attributed to larger LDL I and IIa particles (11,12,22). Based on this assumption, a sample size of 11 participants in each diet group (LC/HF and HC/LF) in a cross-over design was estimated to provide 80% power (alpha = 0.05) to yield detectable differences (G*POWER). For all comparisons (Baseline versus post-Run-In, PS versus Butter) we performed a dependent *t*-test. A Bonferroni correction was applied for the primary comparisons between PS and Butter diets based on seven main outcome variables (i.e., LDL-C, HDL-C, triglycerides, LDL I, LDL II, LDL III, LDL IV) where *p* < 0.007 indicated statistical significance. *p* < 0.05 was used to determine statistical significance for all other secondary outcomes. Effect sizes (Cohen’s d) were calculated for all outcome variables and unadjusted *p*-values included for all primary and secondary outcomes. Statistical analyses were performed using IBM SPSS Statistics for Mac, version 26 (IBM, Armonk, NY, USA). 

## 3. Results

### 3.1. Run-In Period

An equal number of men and women were enrolled and completed the Canola Diet Run-In period in both the LC/HF and HC/LF groups (Table 2). Consistent with a typical response to carbohydrate restriction, the Run-In period resulted in several significant changes in the LC/HF group including a small decrease in body mass and waist circumference, fasting glucose and insulin concentrations, and increase in LDL particles across the size spectrum. The LC/HF Run-In also decreased plasma PL total SFAs, mainly attributed to lower abundance of 16:0 (Table 3). In terms of other PL fatty acids, the LC/HF Run-In decreased 20:2n6, 20:3n6, 20:4n3, 22:4n6, and 22:5n6, whereas 14:1, 17:0, 18:3n3, 20:4n6, and 24:1 were increased. The LC/HF Run-In also affected plasma TG fatty acids including decreased 14:0, 16:1n7, 20:2n6, and 20:3n6, and increased 17:0, 18:0, 18:1n9, 18:3n3, and 20:4n6 (Supplementary Materials Table S3). 

The Run-In period in the HC/LF group resulted in a small decrease in fat mass and HDL-C (Table 2) and affected several PL fatty acids including decreased 18:2n6 and 22:0 and increased 15:0, 17:0, 18:1n9, 20:1n9, 20:4n3, 20:5n3, 22:5n3, 22:6n3, and 24:1 (Table 3). The HC/LF Run-In also increased TG fatty acids including 18:3n3, 20:0, 20:5n3, and 22:5n3 (Supplementary Materials Table S3). 

### 3.2. Butter versus PS

**Figure 2:**
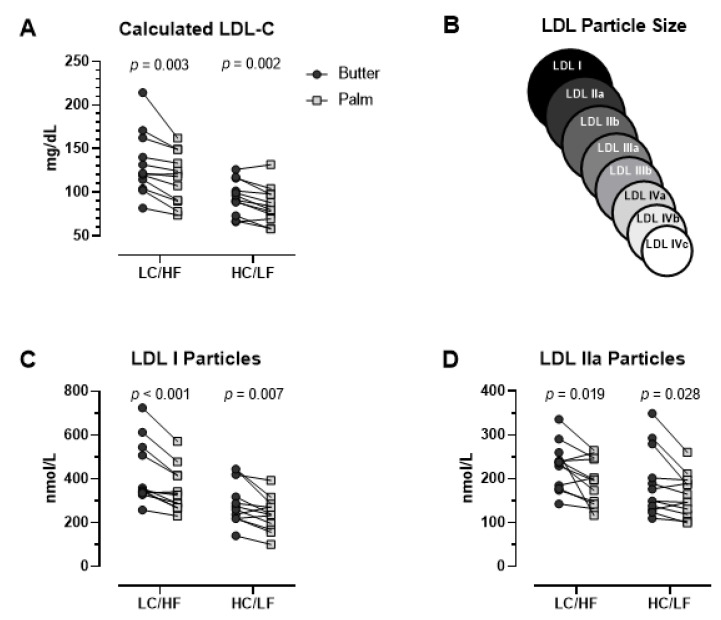
Individual fasting plasma low-density lipoprotein (LDL) responses between Butter and Palm Stearin diets in the low-carbohydrate/high-fat (LC/HF) and high-carbohydrate/low-fat (HC/LF) diet groups. (**A**) LDL-C (calculated), (**B**) LDL size schematic indicating LDL I and IIa as largest particles (**C**) LDL I (ion mobility), and (**D**) LDL IIa (ion mobility). *p*-values based on dependent *t*-tests.

With the exception of a small decrease in percent body fat (0.6%) in the HC/LF group, there were no significant differences between Butter and PS in body mass, glucose, insulin, and standard lipid panels in either group with the notable exception of higher LDL-C in Butter in both the LC/HF and HC/LF groups (Table 4). Compared to PS, LDL-C in Butter was 13.4% higher in the LC/HF group (*p* = 0.003, ES = 0.478) and 10.8% higher in the HC/LF group (*p* = 0.002, ES = 0.456). All but one participant in the LC/HF group and two participants in the HC/LF group had higher LDL-C with Butter (Figure 2A). The higher LDL-C with Butter was attributed primarily to the large LDL I and LDL IIa particles (Table 4 and Figure 2C,D). Compared to PS, LDL I was 19.5% higher on the Butter diet in the LC/HF group (*p* = 0.001, ES = 0.559) and 14.0% higher in the HC/LF group (*p* = 0.007, ES = 0.581). Plasma LDL IIa demonstrated a similar response as LDL I, but small to mid-size LDL species were not affected (Table 4). Individual responses showed that 11 of 12 participants in the LC/HF group and 10 of 12 participants in the HC/LF group had higher large LDL I with Butter (Figure 2C). Oxidized LDL was lower after PS than Butter in the LC/HF group (*p* = 0.011), but there was no difference in HC/LF. 

There were 25 individual plasma PL fatty acids quantified and 14 were different (*p* < 0.05) between Butter and PS in the LC/HF group, but only 2 fatty acids were different in HC/LF (Table 5). In the LC/HF group, the fatty acids that were higher with Butter included 14:0, 17:0, 18:0, 20:2n6, 20:3n6, 20:4n6, 20:4n3, 22:4n6, 22:5n6, 22:5n3, and 24:1, whereas those that were lower included 16:0, 18:2n6, and 20:0. In the HC/LF group, 17:0 and 20:4n6 were higher with Butter than PS.

There were 21 individual plasma TG fatty acids quantified and 8 were different (*p* < 0.05) between Butter and PS in the LC/HF group, but only one fatty acid was different in HC/LF (Supplementary Materials Table S4). In the LC/HF group, the fatty acids that were higher with Butter included 14:0, 15:0, 16:1n7, 17:0, 18:0, 18:3n3, and 22:5n3, whereas 18:2n6 was lower. In the HC/LF group, only 17:0 was higher with Butter than PS.

## 4. Discussion

The primary finding of this randomized, controlled-feeding, cross-over trial in healthy adults with normal cholesterol profiles was that a Butter-based diet increased LDL-C compared to a PS-based diet with no differences in HDL-C or triglycerides. The LDL-C raising effect of Butter relative to PS occurred in the context of diets that were eucaloric, isonitrogenous, and similar total SFA content, suggesting that the food matrix impacted cholesterol metabolism. The higher LDL-C concentrations with Butter was attributed to an increase in larger, cholesterol-rich LDL species, while smaller LDL particles were unaffected. We replicated these findings in two separate cohorts who self-selected to consume either a LC/HF diet or a HC/LF diet. Since each primary fat constituted 40% of the total daily fat intake, the mean absolute amount of butter and PS consumed was 97 and 29 g/day for the LC/HF and HC/LF diets, respectively. Thus, the LDL-C raising effects of Butter relative to PS (on average 11–13%) is consistent across a range of absolute intakes. Although these results support previous work that has established the cholesterol raising response to butter [30], this work extends that knowledge by studying butter in the context of a LC/HF diet. The results are also novel because most studies assessing the cholesterol responses to specific SFA-containing foods have compared them to foods/diets lower in SFA content, as opposed to each other as was the case with Butter versus PS. 

Several features of butter relative to PS, independent of total SFA content, could account for the consistently higher LDL-C concentrations. One factor is that butter has substantially more myristic and lauric acid, whereas the predominant SFA in PS is palmitic acid. Myristic and lauric acids have been shown to increase LDL-C more than palmitic acid [11]. Lower LDL-C with PS may also be due to structural differences with a greater proportion of the SFA bound to the *sn*-1 and *sn*-3 positions, compared to the *sn*-2 position for animal fats like butter. Whether this so-called *sn*-2 hypothesis [12,31] explains the findings here is speculative as we did not determine the positional distribution of Butter and PS. However, assuming the PS was mostly unsaturated at the *sn*-2 position, this may explain the lower plasma LDL-C compared to Butter [12,31]. Finally, because butter contains cholesterol, the Butter-based diets were associated with higher cholesterol intake (77 mg/day for the HC/LF diet and 256 mg/day for the LC/HF diet). Although this slightly higher intake of dietary cholesterol on the Butter diets could translate into an increase in LDL-C, it is probably not the primary driver [32]. 

Further insights into the cholesterol responses to Butter and PS were gleaned by assessing LDL subspecies and oxidized LDL, which showed the higher LDL-C with Butter was attributed to larger LDL I and IIa particles while small species were unchanged. Increased consumption of SFA typically results in higher levels of larger LDL particles, and if substituted for carbohydrate often decreases smaller LDL particles [23,24,25]. Small LDL particles are more highly associated with atherosclerosis and CVD, perhaps as a result of being more prone to oxidation [33,34,35]. There was higher oxidized LDL in Butter than PS (14%, *p* = 0.011) but this only occurred in the LC/HF diet, and it was not associated with any differences in small LDL species. Potential reasons for the higher oxidized LDL with Butter remain speculative but could relate to the presence of antioxidant/anti-inflammatory components in PS such as tocotrienols. 

In order to provide additional insights into potential health implications of Butter and PS diets, we assessed plasma fatty acid composition in PL and TG fractions because they have been shown to predict risk of prediabetes, type 2 diabetes, heart failure and mortality [17,18,19,20,21], whereas free-fatty acids do not. The most notable difference between Butter and PS was higher odd chain SFAs 15:0 and 17:0 in Butter, which is consistent with their primary dietary source being cow’s milk and increased abundance a proxy for dairy fat intake. There were few other differences in PL fatty acids between Butter and PS in the HC/LF diet, whereas the differences were extensive in the LC/HF diet. The individual PL fatty acids that were significantly different between Butter and PS in the LC/HF diet partially reflected the corresponding fatty acid composition of those foods. For example, the linoleic acid content of PS was 4-fold higher than Butter, which likely accounted for the higher abundance in the PS diet, but interesting this only occurred in the LC/HF and not the HC/LF group. Plasma PL arachidonic acid was lower in the PS than Butter diets, which may also reflect higher arachidonic content of Butter. Why some plasma fatty acid differences manifested in the context of LC/HF but not HC/LF remains unclear, but may imply that circulating fatty acid partitioning is sensitized to different SFA-containing foods in the setting of carbohydrate restricted/higher fat diets. 

A strength of this study was the use of controlled feeding periods where all food was provided, which helped manage variability and non-compliance that is common in free-living diet studies. The fact that the primary outcome of increased LDL-C with Butter versus PS was reproduced in the context of two diets varying substantially in macronutrient distribution provides further confidence in the results. The 3-week controlled-feeding Run-In period was important to ensure stabilization of weight and background diet, particularly in the context of the LC/HF diet track since switching to this eating pattern from a habitual mixed diet is associated with robust metabolic adaptations including significant changes in fatty acid composition as evidenced by the highest effect sizes of any outcomes (see Table 2 and Table 3). The results have ‘real-world’ implications since the diets were prepared to reflect typical meal patterns. In lieu of growing evidence questioning the strength of scientific evidence for lowering dietary saturated fat [5] and the popularity of low-carbohydrate diets, understanding how different SFA-containing foods impact circulating lipids as it relates to CVD is important. Weaknesses include a relatively low number of participants, use of PS versus the more commonly consumed palm oil, and an inability to make definitive statements on actual CVD risk considering all the outcomes were surrogate endpoints.

In summary, we present evidence from a controlled-feeding cross-over study in healthy normolipidemic adults in which two commonly consumed SFA-containing foods (butter and palm stearin) demonstrated different effects on cholesterol profiles. Notably butter consistently increased LDL-C more than PS but did so by virtue of larger diameter LDL particles. The increase in larger LDL particles with Butter occurred across a range of intakes in the context of both a LC/HF and HC/LF diet. Overall, these data highlight the importance of the food matrix in determining the downstream biological effects of SFA-containing foods on cardiovascular risk.

## Figures and Tables

**Table 1:** Daily nutrient intakes in the Canola Oil (Run-In) and experimental Butter and Palm Stearin (PS) Diets in the low-carbohydrate/high-fat (LC/HF) and high-carbohydrate/low-fat (HC/LF) diet tracks.

NUTRIENT	LC/HF (*n* = 12)			HC/LF (*n* = 12)		
	Canola	Butter	PS	Canola	Butter	PS
Energy (kcal)	2975			2892		
	(1850–4300)			(1900–4000)		
Protein (g)	136		18%en	129		18%en
	(84–197)			(85–179)		
Carbohydrate (g)	56		8%en	432		60%en
	(35–81)			(284–598)		
Fat (g)	245		74%en	72		22%en
	(152–198)			(47–100)		
Fiber (g)	14			28		
	(9–20)			(19–39)		
Primary Fat Source (g) Canola, Butter, PS	97			29		
	(60–141)			(19–40)		
Saturated fat (g)	74	129	116	17	22	29
	(46–108)	(80–186)	(72–167)	(11–24)	(22–45)	(19–40)
Monounsaturated fat (g)	109	72	83	31	21	24
	(68–157)	(45–105)	(52–121)	(21–43)	(14–29)	(16–33)
Polyunsaturated fat (g)	42	18	32	17	10	11
	(26–60)	(11–26)	(15–34)	(11–18)	(7–14)	(8–16)
Cholesterol (mg)	506	762	506	230	307	230
	(314–731)	(474–1101)	(314–731)	(151–318)	(202–425)	(151–318)

All values reported as Mean (range). Percent energy for each macronutrient is reported as %en. All diets were defined a priori based on diet parameters for each diet period (see methods), but differed between participants based on estimated energy requirements. The primary fats comprised 40% of total fat.

**Table 2:** Baseline and Canola Oil (Run-In) Diet responses in the low-carbohydrate/high-fat (LC/HF) and high-carbohydrate/low-fat (HC/LF) groups.

	LC/HF (*n* = 12)				HC/LF (*n* = 12)			
	Baseline	Canola	*p*-Value	ES	Baseline	Canola	*p*-Value	ES
Sex (Male/Female)	6/6				6/6			
Age (years)	30.9 ± 13.4				31.6 ± 9.7			
Body weight (kg)	72.9 ± 4.1	70.7 ± 4.1	0.000	0.653	72.5 ± 3.6	72.0 ± 3.5	0.125	0.040
Waist circumference (cm)	78.9 ± 2.2	77.0 ± 2.3	0.004	0.252	81.3 ± 2.4	81.7 ± 2.4	0.448	0.050
Hip circumference (cm)	99.6 ± 1.5	98.4 ± 1.7	0.053	0.232	100.3 ± 2.6	99.0 ± 2.3	0.130	0.156
SBP (mmHg)	112 ± 3	110 ± 3	0.277	0.191	110 ± 3	105 ± 3	0.078	0.441
DBP (mmHg)	73 ± 2	71 ± 2	0.036	0.418	71 ± 2	69 ± 2	0.449	0.236
Fat mass (kg)	16.5 ± 1.2	15.5 ± 1.2	0.000	0.237	19.6 ± 2.4	19.4 ± 2.4	0.006	0.081
Lean mass (kg)	53.7 ± 4.5	52.4 ± 4.5	0.006	0.052	50.2 ± 2.8	51.8 ± 2.3	0.906	0.002
% Fat mass	24.6 ± 2.5	24.0 ± 2.6	0.038	0.069	27.7 ± 2.7	26.9 ± 2.9	0.004	0.071
Glucose (mg/dL)	89.1 ± 2.2	82.7 ± 2.1	0.007	0.862	87.7 ± 1.5	84.5 ± 1.8	0.164	0.554
Insulin (μIU/mL)	3.5 ± 0.5	2.4 ± 0.3	0.010	0.762	4.3 ± 0.6	3.7 ± 0.3	0.244	0.321
Triglyceride (mg/dL)	84.9 ± 10.9	85.9 ± 11.1	0.925	0.026	73.5 ± 7.2	77.1 ± 6.8	0.552	0.147
Total cholesterol (mg/dL)	186.9 ± 7.4	186.4 ± 8.2	0.949	0.018	163.1 ± 7.2	151.5 ± 7.2	0.082	0.463
LDL-cholesterol (mg/dL)	91.8 ± 6.1	95.4 ± 5.3	0.510	0.211	88.6 ± 6.1	84.6 ± 5.6	0.383	0.210
HDL-cholesterol (mg/dL) Oxidized LDL (μg/mL)	77.8 ± 6.8 43.3 ± 4.0	73.0 ± 7.1 46.2 ± 3.4	0.117 0.230	0.201 0.226	59.6 ± 2.3 47.9 ± 4.6	51.5 ± 2.2 45.2 ± 4.8	0.000 0.458	1.033 0.166
LDL particles (nmol/L)								
Largest I	245.6 ± 12.1	267.9 ± 21.5	0.299	0.437	246.7 ± 23.1	245.9 ± 17.4	0.958	0.012
IIa	151.7 ± 10.0	175.1 ± 13.0	0.097	0.582	158.6 ± 15.9	171.4 ± 14.5	0.363	0.242
IIb	142.5 ± 12.2	169.5 ± 12.3	0.031	0.636	148.4 ± 16.1	157.1 ± 14.5	0.477	0.164
IIIa	107.5 ± 13.9	135.5 ± 15.9	0.000	0.540	112.2 ± 12.9	111.6 ± 10.7	0.947	0.014
IIIb	43.0 ± 5.0	56.9 ± 10	0.025	0.526	45.2 ± 6.0	43.4 ± 4.4	0.563	0.098
IVa	56.3 ±4.4	71.0 ± 7.8	0.048	0.671	61.5 ± 9.3	59.4 ± 6.5	0.660	0.078
IVb	57.8 ± 4.6	63.2 ± 2.7	0.155	0.422	61.1 ± 8.4	60.8 ± 4.6	0.960	0.012
Smallest IVc	65.2 ± 2.0	69.7 ± 2.5	0.106	0.573	66.5 ± 4.5	68.2 ± 4.7	0.629	0.111

Means ± SEM. Subjects self-selected into LC/HF and HC/LF diet groups. *p*-values are derived from dependent *t*-tests. ES = Effect Size, LDL = low-density lipoprotein, HDL = high-density lipoprotein, SBP = systolic blood pressure, DBP = diastolic blood pressure.

**Table 3:** Baseline and Canola Oil (Run-In) Diet plasma phospholipid fatty acid responses in the low-carbohydrate/high-fat (LC/HF) and high-carbohydrate/low-fat (HC/LF) groups.

	LC/HF (*n* = 12)				HC/LF (*n* = 12)			
Phospholipid (wt%)	Baseline	Canola	*p*-Value	ES	Baseline	Canola	*p*-Value	ES
14:0	0.48 ± 0.05	0.45 ± 0.03	0.574	0.201	0.52 ± 0.03	0.54 ± 0.02	0.415	0.196
14:1	0.17 ± 0.02	0.23 ± 0.02	0.005	0.705	0.21 ± 0.02	0.23 ± 0.02	0.231	0.266
15:0	0.19 ± 0.02	0.21 ± 0.01	0.289	0.422	0.19 ± 0.01	0.22 ± 0.01	0.029	0.849
16:0	26.84 ± 0.51	25.59 ± 0.41	0.016	0.772	26.20 ± 0.47	26.12 ± 0.36	0.767	0.055
16:1w7	0.45 ± 0.11	0.36 ± 0.04	0.257	0.302	0.62 ± 0.11	0.60 ± 0.10	0.732	0.091
17:0	0.40 ± 0.03	0.48 ± 0.01	0.020	1.084	0.42 ± 0.02	0.49 ± 0.02	0.000	1.268
17:1	0.09 ± 0.02	0.11 ± 0.02	0.357	0.333	0.12 ± 0.01	0.11 ± 0.02	0.912	0.175
18:0	11.95 ± 0.31	11.80 ± 0.33	0.630	0.136	12.56 ± 0.36	12.11 ± 0.34	0.111	0.372
18:1w9	10.60 ± 0.45	11.01 ± 0.26	0.521	0.323	10.52 ± 0.27	11.05 ± 0.22	0.032	0.616
18:2w6	23.78 ± 0.93	24.94 ± 0.49	0.142	0.449	23.62 ± 0.54	20.72 ± 0.56	0.000	1.526
18:3w3	0.23 ± 0.04	0.45 ± 0.03	0.003	1.687	0.25 ± 0.04	0.31 ± 0.05	0.220	0.396
20:0	0.20 ± 0.02	0.22 ± 0.02	0.123	0.300	0.23 ± 0.02	0.22 ± 0.02	0.497	0.167
20:1w9	0.16 ± 0.01	0.16 ± 0.01	0.859	0.000	0.16 ± 0.02	0.20 ± 0.01	0.021	0.784
20:2w6	0.41 ± 0.03	0.27 ± 0.01	0.000	1.692	0.38 ± 0.06	0.41 ± 0.02	0.208	0.424
20:3w6	2.95 ± 0.17	1.38 ± 0.08	0.000	3.424	3.04 ± 0.19	3.36 ± 0.23	0.058	0.440
20:4w6	12.86 ± 0.63	14.18 ± 0.41	0.040	0.716	13.02 ± 0.42	14.03 ± 0.50	0.026	0.628
20:4w3	0.07 ± 0.01	0.01 ± 0.00	0.000	2.683	0.04 ± 0.01	0.07 ± 0.01	0.014	0.728
20:5w3	0.60 ± 0.05	0.53 ± 0.04	0.242	0.423	0.49 ± 0.04	0.76 ± 0.08	0.001	1.314
22:0	0.35 ± 0.05	0.37 ± 0.06	0.375	0.114	0.42 ± 0.05	0.38 ± 0.05	0.027	0.222
22:4w6	0.54 ± 0.04	0.45 ± 0.03	0.006	0.740	0.52 ± 0.02	0.57 ± 0.06	0.385	0.328
22:5w6	0.38 ± 0.03	0.25 ± 0.02	0.000	1.506	0.36 ± 0.05	0.37 ± 0.03	0.910	0.073
22:5w3	0.82 ± 0.07	0.77 ± 0.05	0.235	0.225	0.79 ± 0.06	0.94 ± 0.06	0.000	0.731
22:6w3	2.63 ± 0.18	2.80 ± 0.13	0.196	0.305	2.52 ± 0.25	3.15 ± 0.21	0.000	0.791
24:0	0.32 ± 0.04	0.30 ±0.04	0.329	0.154	0.38 ± 0.05	0.35 ±0.05	0.192	0.171
24:1	0.41 ± 0.08	0.52 ± 0.10	0.005	0.358	0.55 ± 0.08	0.67 ± 0.11	0.025	0.360
SFA	40.7 ± 0.4	39.4 ± 0.4	0.005	1.028	40.9 ± 0.2	40.4 ± 0.3	0.115	0.571
MUFA	12.0 ± 0.6	12.5 ± 0.2	0.424	0.340	12.3 ± 0.3	13.0 ± 0.3	0.016	0.702
PUFA	45.4 ± 0.7	46.1 ± 0.3	0.180	0.381	45.1 ± 0.3	44.8 ± 0.5	0.381	0.263

Means ± SEM. *p*-values are derived from dependent *t*-tests. ES = Effect Size. SFA = saturated fatty acids, MUFA = monounsaturated fatty acids, PUFA = polyunsaturated fatty acids.

**Table 4:** Butter versus Palm Stearin (PS) Diet responses in the low-carbohydrate/high-fat (LC/HF) and high-carbohydrate/low-fat (HC/LF) groups.

	LC/HF (*n* = 12)				HC/LF (*n* = 12)			
	Butter	PS	*p*-Value	ES	Butter	PS	*p*-Value	ES
Body weight (kg)	71.0 ± 4.5	70.9 ± 4.6	0.734	0.008	72.1 ± 3.6	71.4 ± 3.5	0.312	0.052
Waist circumference (cm)	77.5 ± 2.6	77.5 ± 2.6	0.908	0.006	80.5 ± 2.1	80.9 ± 1.9	0.756	0.059
Hip circumference (cm)	98.7 ± 1.7	98.4 ± 1.8	0.651	0.054	99.0 ± 2.3	99.0 ± 2.7	0.979	0.002
SBP (mmHg)	110 ± 3	109 ± 2	0.451	0.154	107 ± 3	109 ± 3	0.498	0.129
DBP (mmHg)	72 ± 2	70 ± 2	0.183	0.261	68 ± 2	71 ± 1	0.192	0.534
Fat mass (kg)	15.3 ± 1.0	15.2 ± 1.0	0.637	0.031	19.1 ± 2.5	18.4 ± 2.5	0.037	0.081
Lean mass (kg)	52.9 ± 4.5	53.1 ± 4.6	0.397	0.014	50.4 ± 2.8	50.3 ± 2.7	0.743	0.012
% Fat mass	23.6 ± 2.3	23.4 ± 2.2	0.519	0.026	27.0 ± 2.9	26.4 ± 2.9	0.007	0.064
Glucose (mg/dL)	85.1 ± 2.3	87.1 ± 2.5	0.217	0.237	83.7 ± 2.4	85.3 ± 1.4	0.561	0.244
Insulin (uIU/mL)	2.9 ± 0.3	3.3 ± 0.5	0.278	0.250	3.8 ± 0.3	4.2 ± 0.3	0.323	0.442
Triglyceride (mg/dL)	79.5 ± 7.8	71.3 ± 8.1	0.408	0.299	66.1 ± 6.1	74.1 ± 8.4	0.167	0.315
Total cholesterol (mg/dL)	219.7 ± 13.7	204.7 ± 10.0	0.011	0.362	161.5 ± 7.0	152.8 ± 7.7	0.016	0.341
LDL-cholesterol (mg/dL)	131.9 ± 10.4	116.3 ± 8.4	0.003	0.478	94.3 ± 5.6	85.1 ± 6.0	0.002	0.456
HDL-cholesterol (mg/dL) Oxidized LDL (μg/mL)	71.8 ± 7.8 61.2 ± 4.7	74.1 ± 6.9 53.5 ± 5.0	0.655 0.011	0.088 0.455	54.0 ± 2.0 49.0 ± 5.2	52.9 ± 2.2 50.0 ± 5.5	0.524 0.779	0.147 0.055
LDL particles (nmol/L)								
Largest I	419.9 ± 41.0	351.3 ± 28.9	0.001	0.559	289.7 ± 28.4	238.2 ± 22.5	0.007	0.581
IIa	229.0 ± 15.5	193.5 ± 15.1	0.019	0.670	190.6 ±22.2	162.5 ± 13.9	0.028	0.437
IIb	193.9 ± 14.8	169.4 ± 12.7	0.099	0.512	166.8 ± 18.2	152.9 ± 11.6	0.252	0.262
IIIa	137.5 ± 10.5	130.6 ± 15.0	0.351	0.153	119.0 ± 12.2	112.5 ± 8.2	0.590	0.179
IIIb	51.5 ± 2.7	54.0 ± 7.2	0.625	0.135	47.8 ± 5.2	43.7 ± 3.1	0.350	0.280
IVa	67.2 ± 2.8	66.9 ±4.3	0.937	0.023	64.9 ± 8.5	55.3 ± 4.8	0.079	0.403
IVb	69.2 ± 3.4	65.1 ± 3.9	0.103	0.322	61.4 ± 6.9	56.4 ± 4.7	0.167	0.247
Smallest IVc	81.4 ± 4.3	77.0 ± 3.9	0.313	0.312	67.7 ± 5.1	62.5 ± 2.6	0.216	0.364

Means ± SEM. *p*-values are derived from dependent *t*-tests. ES = Effect Size. LDL = low-density lipoprotein, HDL = high-density lipoprotein, SBP = systolic blood pressure, DBP = diastolic blood pressure.

**Table 5:** Butter versus Palm Stearin (PS) Diet plasma phospholipid fatty acid responses in the low-carbohydrate/high-fat (LC/HF) and high-carbohydrate/low-fat (HC/LF) groups.

	LC/HF (*n* = 12)				HC/LF (*n* = 12)			
Phospholipid (wt%)	Butter	PS	*p*-Value	ES	Butter	PS	*p*-Value	ES
14:0	0.50 ± 0.04	0.42 ± 0.03	0.002	0.614	0.53 ± 0.04	0.52 ± 0.03	0.798	0.091
14:1	0.25 ± 0.04	0.23 ± 0.03	0.591	0.172	0.21 ± 0.03	0.22 ± 0.03	0.688	0.105
15:0	0.23 ± 0.01	0.25 ± 0.03	0.550	0.224	0.30 ± 0.04	0.26 ± 0.04	0.447	0.296
16:0	26.85 ± 0.48	27.62 ± 0.40	0.002	0.508	27.50 ± 0.35	27.83 ± 0.31	0.225	0.287
16:1w7	0.38 ± 0.05	0.32 ± 0.04	0.280	0.408	0.68 ± 0.07	0.65 ± 0.10	0.552	0.103
17:0	0.49 ± 0.01	0.39 ± 0.01	0.000	3.922	0.49 ± 0.02	0.42 ± 0.02	0.000	0.990
17:1	0.10 ± 0.01	0.13 ± 0.02	0.210	0.543	0.19 ± 0.05	0.15 ± 0.02	0.526	0.284
18:0	12.02 ± 0.23	11.15 ± 0.25	0.001	1.046	11.59 ± 0.33	11.68 ± 0.31	0.531	0.081
18:1w9	10.53 ± 0.24	10.30 ± 0.24	0.261	0.279	10.32 ± 0.23	10.61 ± 0.24	0.213	0.358
18:2w6	23.45 ± 0.70	25.69 ± 0.57	0.000	1.010	20.51 ± 0.53	20.73 ± 0.58	0.479	0.114
18:3w3	0.17 ± 0.04	0.12 ± 0.04	0.164	0.385	0.23 ± 0.03	0.25 ± 0.01	0.396	0.275
20:0	0.20 ± 0.03	0.21 ± 0.02	0.044	0.117	0.24 ± 0.02	0.23 ± 0.02	0.558	0.133
20:1w9	0.10 ± 0.01	0.09 ± 0.00	0.335	0.632	0.14 ± 0.01	0.15 ± 0.01	0.515	0.283
20:2w6	0.33 ± 0.01	0.29 ± 0.01	0.000	0.883	0.40 ± 0.03	0.41 ± 0.03	0.542	0.091
20:3w6	2.00 ± 0.08	1.65 ± 0.08	0.000	1.273	3.38 ± 0.25	3.56 ± 0.28	0.242	0.300
20:4w6	14.23 ± 0.37	13.40 ± 0.38	0.002	0.643	13.87 ± 0.46	13.29 ± 0.48	0.037	0.356
20:4w3	0.02 ± 0.00	0.02 ± 0.00	0.014	0.000	0.05 ± 0.02	0.06 ± 0.01	0.654	0.243
20:5w3	0.52 ± 0.04	0.46 ± 0.05	0.204	0.412	0.59 ± 0.07	0.66 ± 0.07	0.244	0.291
22:0	0.35 ± 0.05	0.36 ± 0.05	0.488	0.059	0.42 ± 0.06	0.41 ± 0.05	0.348	0.051
22:4w6	0.59 ± 0.03	0.53 ± 0.03	0.012	0.600	0.57 ± 0.03	0.55 ± 0.02	0.147	0.235
22:5w6	0.29 ± 0.02	0.27 ± 0.02	0.042	0.307	0.42 ± 0.06	0.38 ± 0.05	0.221	0.222
22:5w3	0.92 ± 0.06	0.80 ± 0.05	0.000	0.631	0.92 ± 0.06	0.88 ± 0.06	0.271	0.190
22:6w3	2.63 ± 0.14	2.63 ± 0.11	0.992	0.000	3.15 ± 0.23	3.14 ± 0.24	0.907	0.012
24:0	0.31 ± 0.04	0.31 ± 0.03	0.722	0.000	0.40 ± 0.06	0.37 ± 0.05	0.126	0.157
24:1	0.44 ± 0.09	0.40 ± 0.08	0.033	0.129	0.69 ± 0.10	0.64 ± 0.10	0.188	0.141
SFA	41.0 ± 0.4	40.7 ± 0.3	0.184	0.198	41.5 ± 0.3	41.7 ± 0.3	0.269	0.215
MUFA	11.9 ± 0.2	11.6 ± 0.2	0.117	0.401	12.5 ± 0.2	12.6 ± 0.2	0.618	0.162
PUFA	45.3 ± 0.5	45.9 ± 0.3	0.042	0.462	44.2 ± 0.4	44.0 ± 0.4	0.533	0.125

Means ± SEM. *p*-values are derived from dependent *t*-tests. ES = Effect Size. SFA = saturated fatty acids, MUFA = monounsaturated fatty acids, PUFA = polyunsaturated fatty acids.

## Data Availability

Data described in the manuscript will be made available upon request from the corresponding author.

## Supplementary Materials

The following are available online at https://www.mdpi.com/article/10.3390/nu13061944/s1, Figure S1: CONSORT- Diagram represents participant passage through the study, Table S1: Fatty acid composition of the primary fat sources, Table S2: One day example meal plans for high-carbohydrate, low-fat (A) and low-carbohydrate/high-fat (Panel B) diets; Table S3: Baseline and Canola Oil (Run-In) Diet plasma triglyceride fatty acid responses in the low-carbohydrate/high-fat (LC/HF) and high-carbohydrate/low-fat (HC/LF) groups; Table S4: Butter versus Palm Stearin (PS) Diet plasma triglyceride fatty acid responses in the low-carbohydrate/high-fat (LC/HF) and high-carbohydrate/low-fat (HC/LF) groups.

## Abbreviations

CVD = cardiovascular disease, DNL = de novo lipogenesis, DXA = dual-energy x-ray absorptiometry, EDTA = ethylenediaminetetraacetic acid, HC/LF = high-carbohydrate/low-fat, IDL = intermediate-density lipoprotein, LC/HF = low-carbohydrate/high-fat, MUFA, monounsaturated fatty acids, PL = phospholipids, PS = Palm Stearin, PUFA = polyunsaturated fatty acids, SFA = saturated fatty acids, TG = triglycerides.
