# Supplementary material for: Effects of Palm Stearin versus Butter in the Context of Low-Carbohydrate/High-Fat and High-Carbohydrate/Low-Fat Diets on Circulating Lipids in a Controlled Feeding Study in Healthy Humans
Source: Nutrients. 2021 Jun 5;13(6):1944. doi: 10.3390/nu13061944 (PMC8226735; doi:10.3390/nu13061944)
Supplement: Supplementary file 1 [file nutrients-13-01944-s001.zip › nutrients-1204574-supplementary.pdf]

Online Supplementary Materials

Hyde et al. Effects of palm stearin versus butter in the context of low-carbohydrate/high-fat and high-carbohydrate/low-fat diets on circulating lipids in a controlled feeding study in healthy humans.

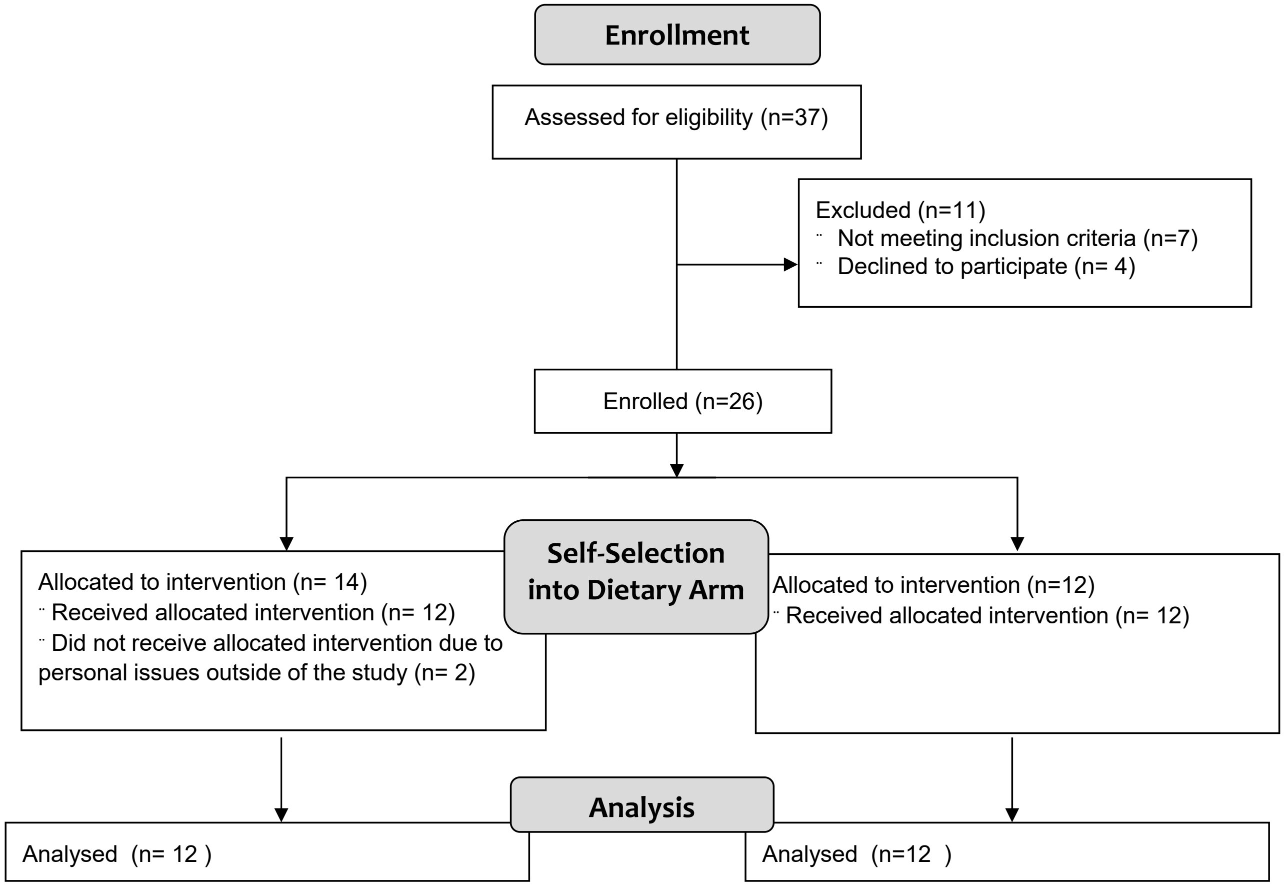

Supplementary Figure S1. CONSORT- Diagram represents participant passage through the study.

Supplementary Table S1. Fatty acid composition of the primary fat sources.

	Canola	Butter	Palm
Kcal	886	698	895
Total PRO	0.26	0.74	<0.10
Total CHO	1.4	7.4	1.1
Kcals from fat	879	666	890
SFA	6.68	48.30	48.50
Total Cis Unsaturated FA	86.40	18.40	45.80
MUFA	63.70	15.90	36.70
PUFA	22.70	2.48	9.08
Trans	0.36	3.41	0.15
O3	7.75	0.34	0.18
O6	16.00	2.25	9.31
O9	63.20	13.80	37.50
Total FA	97.70	74.00	98.90
4:0	<0.07	2.90	<0.07
6:0	<0.07	1.73	<0.07
8:0	<0.07	1.00	<0.07
10:0	<0.07	2.31	<0.07
12:0	<0.07	2.62	0.17
14:0	<0.07	8.12	1.03
14:1	<0.07	0.72	<0.07
15:0	<0.07	0.84	<0.07
16:0	4.22	23.60	44.90
16:1	0.21	1.12	0.15
17:0	<0.07	0.43	0.09
18:0	1.80	7.54	4.33
18:1n9	61.80	13.80	37.40
18:2w6	16.00	2.05	9.31
18:3w3	7.75	0.34	0.18
20:0	0.56	0.10	0.35
20:1	1.28	<0.07	0.15

Note: Numbers represent the amount per 100g of tested product. Fatty acid species not listed were below limits of detection from Covance.

Supplementary Table S2. One day example meal plans for high-carbohydrate, low-fat (A) and low-carbohydrate/high-fat (Panel B) diets.

A

High Carbohydrate		MUFA			PUFA			SFA	
		Canola	Butter	Palm	Canola	Butter	Palm	Canola	Butter
Total (g)		28.7	19.0	21.9	14.2	7.9	9.4	12.3	26.8
Breakfast (Oatmeal)									
Quick oats	(50.0 g)	1.3	1.3	1.3	1.3	1.3	1.3	0.6	0.6
Peanut butter	(16.0 g)	4.1	4.1	4.1	2.0	2.0	2.0	1.6	1.6
Cinnamon	(2.0 g)	0.0	0.0	0.0	0.0	0.0	0.0	0.0	0.0
Brown sugar	(15.0 g)	0.0	0.0	0.0	0.0	0.0	0.0	0.0	0.0
Skim milk	(245.0 g)	0.1	0.1	0.1	0.0	0.0	0.0	0.1	0.1
Orange juice	(260.0 g)	0.1	0.1	0.1	0.1	0.1	0.1	0.1	0.1
Morning Snack									
Grape juice	(260.0 g)	0.0	0.0	0.0	0.1	0.1	0.1	0.1	0.1
Applesauce	(250.0 g)	0.0	0.0	0.0	0.1	0.1	0.1	0.0	0.0
Rice cakes	(30.0 g)	0.0	0.0	0.0	0.0	0.0	0.0	0.0	0.0
Lunch (Chicken Salad)									
Chicken breast	(85.0 g)	0.9	0.9	0.9	0.6	0.6	0.6	0.7	0.7
Bacon	(9.0 g)	1.4	1.4	1.4	0.6	0.6	0.6	1.1	1.1
Romaine lettuce	(90.0 g)	0.0	0.0	0.0	0.1	0.1	0.1	0.0	0.0
Cherry tomatoes	(45.0 g)	0.0	0.0	0.0	0.0	0.0	0.0	0.0	0.0
Cucumber	(20.0 g)	0.0	0.0	0.0	0.0	0.0	0.0	0.0	0.0
Red bell pepper	(37.3 g)	0.0	0.0	0.0	0.0	0.0	0.0	0.0	0.0
Mozzarella cheese	(20.0 g)	0.9	0.9	0.9	0.1	0.1	0.1	2.0	2.0
Fat	(17.8-22.0)	11.3	4.6	6.6	5.0	0.7	1.7	1.3	11.3
Mrs. Dash seasoning	(0.5 g)	0.0	0.0	0.0	0.0	0.0	0.0	0.0	0.0
Croutons	(10.0 g)	0.0	0.0	0.0	0.0	0.0	0.0	0.0	0.0
Jello	(110.0 g)	0.0	0.0	0.0	0.0	0.0	0.0	0.0	0.0
Mandarin oranges	(150.0 g)	0.0	0.0	0.0	0.0	0.0	0.0	0.0	0.0
Afternoon Snack									
Blueberries	(40.0 g)	0.0	0.0	0.0	0.1	0.1	0.1	0.0	0.0
Granola	(50.0 g)	1.0	1.0	1.0	1.0	1.0	1.0	0.5	0.5
Vanilla yogurt	(100.0 g)	0.0	0.0	0.0	0.0	0.0	0.0	0.0	0.0
Dinner (BBQ Meatballs)									
Ground beef	(125.0 g)	2.0	2.0	2.0	0.3	0.3	0.3	2.3	2.3
Bread crumbs	(20.0 g)	0.2	0.2	0.2	0.4	0.4	0.4	0.2	0.2
Onion	(5.0 g)	0.0	0.0	0.0	0.0	0.0	0.0	0.0	0.0
Skim milk	(15.0 g)	0.0	0.0	0.0	0.0	0.0	0.0	0.0	0.0
Egg beaters	(12.5 g)	0.0	0.0	0.0	0.0	0.0	0.0	0.0	0.0
BBQ sauce	(60.0 g)	0.0	0.0	0.0	0.0	0.0	0.0	0.0	0.0
Russet potato	(200.0 g)	0.0	0.0	0.0	0.1	0.1	0.1	0.1	0.1
Skim milk	(12.0 g)	0.0	0.0	0.0	0.0	0.0	0.0	0.0	0.0
Fat	(5.6-7.0 g)	3.6	1.5	2.1	1.6	0.2	0.5	0.4	3.6
Garlic powder	(0.1 g)	0.0	0.0	0.0	0.0	0.0	0.0	0.0	0.0
Parmesan cheese	(5.0 g)	0.4	0.4	0.4	0.1	0.1	0.1	0.8	0.8
Fat	(2.4-3.0 g)	1.5	0.6	0.9	0.7	0.1	0.2	0.2	1.5

B

Low Carbohydrate		MUFA			PUFA			SFA		
		Canola	Butter	Palm	Canola	Butter	Palm	Canola	Butter	Palm
Total (g)		77.5	53.6	60.7	24.9	9.2	12.8	55.6	91.5	82.5
Breakfast (Scramble)										
Spinach	(39.0 g)	0.0	0.0	0.0	0.0	0.0	0.0	0.0	0.0	0.0
Bacon	(30.0 g)	5.2	5.2	5.2	1.9	1.9	1.9	4.0	4.0	4.0
Fat	(8.1-10.0 g)	5.1	2.1	3.0	2.3	0.3	0.8	0.6	5.1	4.0
Egg beaters	(125.0 g)	0.0	0.0	0.0	0.0	0.0	0.0	0.0	0.0	0.0
Heavy cream	(30.0 g)	3.2	3.2	3.2	0.4	0.4	0.4	6.9	6.9	6.9
Fat	(8.1-10.0 g)	5.1	2.1	3.0	2.3	0.3	0.8	0.6	5.1	4.0
Cheddar cheese	(28.4 g)	2.4	2.4	2.4	0.4	0.4	0.4	5.5	5.5	5.5
Morning Snack										
Pepperoni	(28.0 g)	4.8	4.8	4.8	1.0	1.0	1.0	4.2	4.2	4.2
Cheddar cheese	(28.4 g)	2.4	2.4	2.4	0.4	0.4	0.4	5.5	5.5	5.5
Heavy cream	(30.0 g)	3.2	3.2	3.2	0.4	0.4	0.4	6.9	6.9	6.9
Chicken bouillon	(5.0 g)	0.0	0.0	0.0	0.0	0.0	0.0	0.0	0.0	0.0
Fat	(17.8-22.0 g)	11.3	4.6	6.6	5.0	0.7	1.7	1.3	11.3	8.8
Lunch (Chicken Salad)										
Chicken breast	(70.0 g)	0.7	0.7	0.7	0.5	0.5	0.5	0.6	0.6	0.6
Bacon	(16.0 g)	2.5	2.5	2.5	1.0	1.0	1.0	1.9	1.9	1.9
Romaine lettuce	(72.0 g)	0.0	0.0	0.0	0.1	0.1	0.1	0.0	0.0	0.0
Cherry tomatoes	(45.0 g)	0.0	0.0	0.0	0.0	0.0	0.0	0.0	0.0	0.0
Cucumber	(20.0 g)	0.0	0.0	0.0	0.0	0.0	0.0	0.0	0.0	0.0
Red bell pepper	(37.3 g)	0.0	0.0	0.0	0.0	0.0	0.0	0.0	0.0	0.0
Cheddar cheese	(30.0 g)	2.5	2.5	2.5	0.4	0.4	0.4	5.8	5.8	5.8
Fat	(24.3-30.0 g)	15.4	6.3	9.0	6.8	0.9	2.3	1.8	15.4	12.0
Ranch seasoning	2.0 g)	0.0	0.0	0.0	0.0	0.0	0.0	0.0	0.0	0.0
SF Jello	(89.0 g)	0.0	0.0	0.0	0.0	0.0	0.0	0.0	0.0	0.0
Afternoon Snack										
Red bell pepper	(74.5 g)	0.0	0.0	0.0	0.1	0.1	0.1	0.0	0.0	0.0
Ranch seasoning	(1.9 g)	0.0	0.0	0.0	0.0	0.0	0.0	0.0	0.0	0.0
Sour cream	(29.0 g)	1.5	1.5	1.5	0.2	0.2	0.2	3.3	3.3	3.3
Fat	(5.7-7.0 g)	3.6	1.5	2.1	1.6	0.2	0.5	0.4	3.6	2.8
Dinner (Meatball Parm)										
Ground beef	(113.0 g)	10.0	10.0	10.0	0.6	0.6	0.6	8.6	8.6	8.6
Egg beaters	(20.0 g)	0.0	0.0	0.0	0.0	0.0	0.0	0.0	0.0	0.0
Italian seasoning	(0.2 g)	0.0	0.0	0.0	0.0	0.0	0.0	0.0	0.0	0.0
Garlic powder	(0.2 g)	0.0	0.0	0.0	0.0	0.0	0.0	0.0	0.0	0.0
Onion powder	0.2 g)	0.0	0.0	0.0	0.0	0.0	0.0	0.0	0.0	0.0
Marinara sauce	(63.0 g)	1.8	1.8	1.8	0.8	0.8	0.8	0.5	0.5	0.5
Fat	(8.1-10.0 g)	5.1	2.1	3.0	2.3	0.3	0.8	0.6	5.1	4.0
Mozzarella cheese	(42.5 g)	1.9	1.9	1.9	0.2	0.2	0.2	4.3	4.3	4.3
Broccoli	(95.0 g)	0.0	0.0	0.0	0.1	0.1	0.1	0.0	0.0	0.0
Parmesan cheese	(5.0 g)	0.4	0.4	0.4	0.1	0.1	0.1	0.8	0.8	0.8
Fat	(9.7-12.0 g)	6.2	2.5	3.6	2.7	0.4	0.9	0.7	6.2	4.8

Supplemental Table S3. Baseline and Canola Oil (Run-In) Diet plasma triglyceride fatty acid responses in the low-carbohydrate/high-fat (LC/HF) and high-carbohydrate/low-fat (HC/LF) groups.

	LC/HF (n=12)				HC/LF (n=12)			
	**Baseline**	**Canola**	***P-value***	**ES**	**Baseline**	**Canola**	***P-value***	**ES**
12:0	0.05 ± 0.03	0.09 ± 0.05	0.196	0.287	0.03 ± 0.03	0.02 ± 0.02	0.457	0.117
14:0	1.79 ± 0.20	1.18 ± 0.10	0.035	1.109	1.53 ± 0.12	1.66 ± 0.14	0.250	0.292
14:1	0.14 ± 0.04	0.07 ± 0.02	0.111	0.691	0.13 ± 0.02	0.16 ± 0.03	0.368	0.331
15:0	0.32 ± 0.02	0.32 ± 0.02	0.951	0.000	0.29 ± 0.03	0.31 ± 0.02	0.538	0.226
16:0	22.96 ± 0.47	22.66 ± 0.60	0.647	0.161	22.18 ± 0.37	21.82 ± 1.04	0.689	0.134
16:1w7	4.19 ± 0.40	2.00 ± 0.17	0.000	2.082	4.20 ± 0.35	4.55 ± 0.36	0.114	0.285
17:0	0.37 ± 0.02	0.44 ± 0.03	0.037	0.822	0.41 ± 0.03	0.41 ± 0.03	0.771	0.000
18:0	2.81 ± 0.20	3.95 ± 0.22	0.000	1.560	3.00 ± 0.13	2.80 ± 0.11	0.231	0.487
18:1w9	36.40 ± 1.11	40.55 ± 0.70	0.017	1.288	37.53 ± 0.56	38.69 ± 0.69	0.098	0.532
18:2w6	22.26 ± 1.37	20.19 ± 0.84	0.227	0.526	23.19 ± 0.51	21.09 ± 0.96	0.064	0.791
18:3w3	1.62 ± 0.15	2.29 ± 0.19	0.000	1.138	1.42 ± 0.10	1.79 ± 0.17	0.010	0.763
20:0	0.22 ± 0.07	0.23 ± 0.05	0.869	0.047	0.09 ± 0.04	0.13 ± 0.05	0.038	0.264
20:1w9	0.32 ± 0.05	0.24 ± 0.03	0.075	0.549	0.30 ± 0.03	0.30 ± 0.02	0.872	0.000
20:2w6	0.36 ± 0.04	0.18 ± 0.02	0.000	1.579	0.32 ± 0.03	0.32 ± 0.05	0.926	0.000
20:3w6	0.40 ± 0.02	0.20 ± 0.01	0.000	3.621	0.46 ± 0.03	0.47 ± 0.03	0.599	0.091
20:4w6	1.61 ± 0.15	2.13 ± 0.13	0.004	1.092	1.66 ± 0.12	1.66 ± 0.09	0.982	0.000
20:5w3	0.20 ± 0.04	0.15 ± 0.02	0.289	0.452	0.12 ± 0.02	0.21 ± 0.04	0.010	0.813
22:4w6	0.24 ± 0.02	0.18 ± 0.03	0.194	0.705	0.19 ± 0.02	0.23 ± 0.01	0.084	0.560
22:5w6	0.15 ± 0.03	0.14 ± 0.02	0.797	0.105	0.15 ± 0.03	0.16 ± 0.02	0.857	0.113
22:5w3	0.40 ± 0.06	0.37 ± 0.05	0.586	0.157	0.33 ± 0.02	0.40 ± 0.04	0.024	0.713
22:6w3	0.43 ± 0.08	0.54 ± 0.08	0.096	0.407	0.32 ± 0.06	0.33 ± 0.06	0.774	0.048
SFA	28.5 ± 0.5	28.9 ± 0.5	0.595	0.209	27.6 ± 0.4	27.2 ± 1.2	0.715	0.129
MUFA	41.6 ± 1.4	43.2 ± 0.7	0.342	0.416	42.5 ± 0.6	44.2 ± 0.6	0.015	0.811
PUFA	28.1 ± 1.7	26.6 ± 0.9	0.402	0.339	28.4 ± 0.6	27.0 ± 1.0	0.230	0.512

Values are expressed as wt%, Means ± SEM. *P*-values are derived from dependent t-tests. ES=Effect Size.

Supplemental Table S4. Butter versus Palm Stearin (PS) Diet plasma triglyceride fatty acid responses in the low-carbohydrate/high-fat (LC/HF) and high-carbohydrate/low-fat (HC/LF) groups.

	LC/HF (n=12)				HC/LF (n=12)			
	**Butter**	**PS**	***P-value***	**ES**	**Butter**	**PS**	***P-value***	**ES**
12:0	0.05 ± 0.03	0.04 ± 0.03	0.643	0.091	0.00 ± 0.00	0.09 ± 0.05	0.121	0.747
14:0	1.69 ± 0.16	1.16 ± 0.08	0.001	1.189	1.71 ± 0.18	1.65 ± 0.19	0.652	0.094
14:1	0.18 ± 0.03	0.12 ± 0.06	0.258	0.372	0.14 ± 0.04	0.14 ± 0.04	0.988	0.000
15:0	0.44 ± 0.03	0.30 ± 0.03	0.004	1.268	0.37 ± 0.02	0.31 ± 0.02	0.052	0.849
16:0	27.36 ± 0.47	28.43 ± 0.42	0.062	0.694	24.28 ± 0.51	24.61 ± 0.72	0.486	0.153
16:1w7	2.50 ± 0.10	2.14 ± 0.10	0.011	1.058	4.51 ± 0.31	4.41 ± 0.29	0.652	0.095
17:0	0.54 ± 0.03	0.45 ± 0.04	0.000	0.815	0.49 ± 0.04	0.40 ± 0.03	0.001	0.656
18:0	4.84 ± 0.27	4.07 ± 0.19	0.004	0.950	3.01 ± 0.16	2.91 ± 0.22	0.518	0.149
18:1w9	37.04 ± 0.70	37.71 ± 0.61	0.211	0.296	36.74 ± 0.44	37.30 ± 0.56	0.228	0.322
18:2w6	17.30 ± 0.82	19.30 ± 0.71	0.014	0.750	20.38 ± 0.74	20.46 ± 1.20	0.901	0.023
18:3w3	1.08 ± 0.07	0.96 ± 0.07	0.005	0.490	1.28 ± 0.07	1.20 ± 0.06	0.248	0.332
20:0	0.17 ± 0.03	0.11 ± 0.02	0.062	0.663	0.09 ± 0.04	0.10 ± 0.04	0.403	0.069
20:1w9	0.26 ± 0.05	0.25 ± 0.05	0.560	0.057	0.33 ± 0.05	0.30 ± 0.03	0.286	0.201
20:2w6	0.21 ± 0.02	0.17 ± 0.03	0.159	0.470	0.36 ± 0.05	0.33 ± 0.04	0.313	0.181
20:3w6	0.18 ± 0.02	0.17 ± 0.02	0.772	0.133	0.47 ± 0.04	0.46 ± 0.03	0.831	0.083
20:4w6	2.03 ± 0.09	1.96 ± 0.13	0.472	0.179	1.79 ± 0.08	1.72 ± 0.05	0.366	0.299
20:5w3	0.60 ± 0.49	0.07 ± 0.02	0.304	0.438	0.20 ± 0.04	0.18 ± 0.02	0.580	0.192
22:4w6	0.22 ± 0.03	0.21 ± 0.03	0.883	0.111	0.25 ± 0.02	0.23 ± 0.02	0.417	0.307
22:5w6	0.17 ± 0.02	0.14 ± 0.02	0.160	0.429	0.18 ± 0.05	0.17 ± 0.03	0.876	0.077
22:5w3	0.39 ± 0.06	0.31 ± 0.05	0.028	0.420	0.36 ± 0.03	0.39 ± 0.04	0.447	0.228
22:6w3	0.46 ± 0.06	0.35 ± 0.06	0.121	0.579	0.39 ± 0.06	0.32 ± 0.05	0.369	0.364
SFA	28.5 ± 0.5	28.9 ± 0.5	0.595	0.209	27.6 ± 0.4	27.2 ± 1.2	0.715	0.129
MUFA	41.6 ± 1.4	43.2 ± 0.7	0.342	0.416	42.5 ± 0.6	44.2 ± 0.6	0.015	0.811
PUFA	28.1 ± 1.7	26.6 ± 0.9	0.402	0.339	28.4 ± 0.6	27.0 ± 1.0	0.230	0.512

Values are expressed as wt%, Means ± SEM. *P*-values are derived from dependent t-tests. ES=Effect Size.
